# Older patients’ experiences of two exercise programmes in a geriatric ward: a qualitative study

**DOI:** 10.1007/s41999-026-01569-w

**Published:** 2026-08-05

**Authors:** Linda Sandberg, Nathalie Frisendahl, Christina Sandlund, Anne-Marie Boström, Anna-Karin Welmer

**Affiliations:** 1 Division of Physiotherapy, Department of Neurobiology, Care Sciences and Society, Karolinska Institutet, Stockholm, Sweden; 2 Department of Geriatric Medicine, Capio Geriatrik Dalen, Capio Elderly and Mobile Care, Stockholm, Sweden; 3 Academic Primary Health Care Centre, Region Stockholm, Stockholm, Sweden; 4 Division of Nursing and Division of Family Medicine and Primary Care, Department of Neurobiology, Care Sciences and Society, Karolinska Institutet, Stockholm, Sweden; 5 Research and Development Unit, Stockholms Sjukhem, Stockholm, Sweden; 6 Aging Research Center, Department of Neurobiology, Care Sciences and Society, Karolinska Institutet, Stockholm, Sweden; 7 Women’s Health and Allied Health Professionals Theme, Karolinska University Hospital, Stockholm, Sweden

**Keywords:** Exercise programmes, Physical activity, Hospitalisation, Older adults, Geriatric

## Abstract

**Purpose:**

Older adults hospitalised for acute illness are highly vulnerable to functional decline, often exacerbated by inactivity. Although exercise interventions may reduce this risk, uncertainty remains regarding how such programmes should be designed and tailored for frail patients. Embedded within the PREV_FUNC randomized controlled trial, this study explored older patients’ experiences of two in-hospital exercise programmes.

**Methods:**

This qualitative study was conducted at a geriatric hospital in Stockholm, Sweden. Using an inductive approach within a constructivist paradigm, semi-structured interviews were conducted with 24 patients aged ≥ 75 years who had participated in either a comprehensive or a simple exercise programme. Data were analysed inductively using reflexive thematic analysis.

**Results:**

One overarching theme emerged: Structured exercise disrupted inactivity and sparked hope for a turning point. This theme comprised four subthemes: Exercise awakens hope for improvement; Getting out of bed can be challenging; Overall confidence in structured exercise; and Support from the physiotherapist is crucial.

**Conclusion:**

From the patients’ perspective, the exercise programmes helped break periods of inactivity and fostered hope for recovery. These findings suggest that in-hospital exercise may support physical activity in geriatric wards, although individual adaptation and support remain essential.

**Supplementary Information:**

The online version contains supplementary material available at https://doi.org/10.1007/s41999-026-01569-w.

## Key summary points

**Aim:**

To explore older patients’ experiences of participating in exercise programmes during hospitalisation in geriatric care.

**Findings:**

Structured exercise helped patients break prolonged inactivity during hospitalisation and counterbalance illness-related physical decline, fostering hope and belief in recovery. Physiotherapists played an important motivational role.

**Message:**

The exercise programmes, with individual adaptation and support, were described as disrupting inactivity and sparking hope for a turning point among older patients in geriatric care.

**Supplementary Information:**

The online version contains supplementary material available at https://doi.org/10.1007/s41999-026-01569-w.

## Introduction

Acutely hospitalised older adults are often frail and are at high risk of functional decline. Nearly half of adults aged 65 years and older experience some loss of physical function following acute hospitalisation, and many fail to fully regain their pre-hospital level of functioning [1–3]. Such decline is associated with loss of independence, reduced well-being, readmission, institutionalisation, and even increased mortality [1–3].

Hospitalisation itself can accelerate this deterioration. Prolonged bed rest and inactivity contribute to rapid muscle loss and exacerbate frailty and sarcopenia [2]. Low levels of mobility during hospital stays are strongly associated with in-hospital acquired disability and long-term adverse outcomes [2, 3 ]. Furthermore, patients have highlighted that contextual aspects, such as the hospital environment and the availability of staff, influence their opportunities for physical activity [4].

Exercise interventions delivered during hospitalisation have demonstrated promising effects in preventing or mitigating functional decline among older adults. Both comprehensive, multicomponent programmes—typically incorporating strength, balance, and mobility exercises—and simpler programmes involving only a few targeted activities have shown benefits on physical function and independence at discharge [5, 6 ]. However, uncertainty remains regarding how such programmes should be designed and tailored to meet the diverse needs and capacities of older patients with varying degrees of frailty [7, 8 ]. In particular, little is known about how older patients themselves experience different types of in-hospital exercise programmes and how these experiences influence their engagement during hospitalisation.

Previous research has shown that older adults often describe exercise as providing not only physical, but also mental and social benefits, which may enhance motivation and adherence [9–11]. Global recommendations emphasise that physical activity is fundamental for health across the lifespan, with particular importance in later life to counteract sarcopenia and support functional independence [9]. Importantly, these benefits are most likely achieved when programmes are adapted to individual preferences, abilities, and contexts [9–11]. Understanding patients’ perspectives is, therefore, essential for evaluating the acceptability, feasibility, and perceived value of different in-hospital exercise approaches, and for advancing person-centered and sustainable care strategies [12, 13 ].

Since 2022, the three-armed *Preventing Functional Decline in Acutely Hospitalised Older adults* (PREV_FUNC) Randomised Controlled Trial (RCT) has been conducted in Stockholm, Sweden [7, 8 ]. The trial evaluates the effect of two in-hospital multicomponent exercise programmes inspired by Ortiz-Alonso et al., and Martinéz-Velilla et al. [5, 6 ]: (1) a comprehensive programme consisting of mobility, balance, and strength exercises [5], and (2) a simpler programme consisting of walking and sit-to-stand exercises, compared with usual care [6, 8 ]. A pilot study of the trial, using survey data from 33 participants demonstrated feasibility and indicated that the exercise sessions were perceived as meaningful and motivating, with staff support playing a key role in facilitating participation. However, some participants expressed a need for greater variation, and survey data provided limited insight into the deeper experiences, meanings, and concerns associated with participation [7]. These findings highlighted the need for qualitative exploration to better understand how hospitalised older adults perceive and engage with in-hospital exercise interventions.

To date, there remains a lack of in-depth qualitative knowledge about how hospitalised older patients experience structured exercise programmes during acute hospital care, particularly within RCTs evaluating different exercise approaches. Therefore, the aim of this study was to explore and gain a deeper understanding of older patients’ experiences of the two in-hospital exercise programmes within the PREV_FUNC trial.

## Methods

### Study design

This qualitative study was conducted alongside the PREV_FUNC trial and completed prior to the analysis of the RCT outcomes. Using an inductive approach [14], the interview data were analysed through reflexive thematic analysis [15]. A constructivist research paradigm was adopted to guide our study, recognising that reality is subjective and constructed through social interactions. This perspective guided both the data collection and researchers’ analysis, acknowledging that meaning is constructed through the interaction between participants and researchers [16].

The reporting of this study followed the Consolidated criteria for reporting qualitative research (COREQ) (Online Appendix 1) [17].

### Setting and participants

The PREV_FUNC trial was conducted at three geriatric hospitals in Stockholm, Sweden. The present interview study was carried out in one of these hospitals and included participants from three geriatric wards. Only participants allocated to the intervention groups of the PREV_FUNC trial were eligible for inclusion in this interview study. Each ward was staffed by a multiprofessional team, and the median length of in-hospital stay was 7 days. Patients were admitted either directly from their homes or via the emergency department.

Eligibility for participation in the PREV_FUNC included age from 75 years or older, the ability to stand up from a sitting position independently or with minimal assistance, and sufficient ability to communicate and collaborate with the research staff. Exclusion criteria were inability to follow instructions, medical condition contraindicating exercise, residence in care homes, or previous participation in the trial. An overview of the two in-hospital exercise programmes is shown in Table 1.

**Table 1:** An overview of the two in-hospital exercise programmes

The Simple exercise programme	The Comprehensive exercise programme
The intervention consists of up to four daily sessions (a total duration of 20–30 min/day) including sit-to stand exercises and walking along the corridor of the ward	The intervention consists of a morning and an afternoon session (a total duration of 40 min/day) The morning session includes individualised supervised progressive resistance, and balance and walking exercise tailored to each participant’s capacity. The resistance exercise includes using weight cuffs. Balance and gait exercises include line walking, stepping practice and walking with small obstacles The afternoon session consists of functional exercises, such as knee extension and flexion, hand exercise with a ball, and daily walking exercise

Participants in this interview study were recruited between March and October 2024 from the intervention groups of the PREV_FUNC trial. Purposive sampling was used based on age, sex, and group allocation (*Simple* or *Comprehensive exercise programme*) to capture a diverse range of experiences and perspectives [18]. Twenty-five eligible patients were invited to participate throughout the recruitment period at the end of their hospital stay. Recruitment ceased when the research team judged that the interviews provided sufficient information to address the study aim. Twenty-four patients consented to participate; one declined participation, and no reason for declining was recorded

### Data collection

To explore and gain a deeper understanding of participants’ experiences of performing the exercise sessions, interviews were conducted at the end of their hospital stay. Data were collected using a semi-structured interview guide (Online Appendix 2) developed for this study. The guide comprised open-ended questions that initially addressed participants’ previous exercise habits, followed by questions focusing on their experiences of engaging in exercise sessions during the current hospital stay. Each interview ended by asking the patient to reflect on their thoughts about continuing exercise at home after discharge. Follow-up prompts, such as “Can you give an example?”, “Can you tell me more?”, and “What does that mean to you?”, were used to encourage elaboration of participants’ responses. The interview guide was pre-tested with one participant. As only minor linguistic adjustments were made and no substantial revisions were required, the meaning of the interview guide remained unchanged, and this interview was included in the analysis.

All interviews were conducted in Swedish by LS and field notes were taken. Data collection continued until the material was considered information-rich and sufficient to capture recurring patterns of meaning. The interviews took place in a quiet, private room within the hospital and lasted between 8 and 47 min (mean duration: 28 min). All interviews were audio-recorded and transcribed verbatim. Most transcripts were prepared by a professional transcription service, while six interviews were transcribed by members of the research team. The text was validated against field notes and the audio recordings by LS through a line-by-line accuracy check. Minor mishearings were corrected, and contextual details noted during the interview were added when relevant. No substantive content was missing or altered during this verification process.

The data to describe the participants was obtained from the PREV_FUNC trial. Background information included age, sex, cohabitation status, and main cause of admission. Frailty was assessed using the Clinical Frailty Scale (CFS; range 1–9), with higher scores indicating a higher degree of frailty [19]. Baseline physical performance was assessed using the Short Physical Performance Battery (SPPB; range 0–12), with higher scores indicating better performance [20]. Data on length of hospitalisation and number of completed exercise sessions were also obtained.

### Data analysis

We conducted a reflexive thematic analysis [15] to identify, analyse, and report patterns within the interview data. The analysis followed a recursive process of coding, theme development, and writing, allowing for deep engagement with the data.

To initiate the analytic process, LS and NF each conducted an independent analysis of the same initial interviews. This was not done for double-checking purposes, but to allow both researchers to explore early patterns and reflect on their interpretative positions. LS and NF then had regular meetings for in-depth discussions of these interviews to compare insights, examine differences in interpretation, and establish a shared analytic direction. Following this initial phase, they generated initial codes capturing features of the data relevant to the research aim. The remaining interviews were divided between LS and NF for coding, followed by iterative joint review sessions where they cross-checked each other’s coding and discussed whether any potentially meaningful aspects had been overlooked or required further clarification. These codes were subsequently organised into potential themes by identifying patterns and relationships across the dataset. The preliminary themes were reviewed and refined together with the whole research group in relation to the coded data and the dataset as a whole to ensure they accurately represented participants’ experiences and perspectives. Each theme was then defined and named to clearly convey its core meaning. The whole research group collaboratively integrated the themes into a coherent narrative, supported by illustrative quotations from the interviews. Throughout the process, the whole research group worked reflexively, moving back and forth between transcripts, codes, and developing themes.

### Reflexivity

The analytical process was iterative, involving continuous comparison of data and repeated refinement of themes through discussions among all members of the research team. Throughout the research process, all authors maintained a reflexive stance, acknowledging our professional positions and potential biases. As an all-female team with extensive experience in geriatric and rehabilitation care, we share a professional understanding of the importance of movement and physical activity in later life. We recognised that this shared orientation could influence how we interpreted participants’ accounts. To address this, we worked deliberately to remain grounded in the interview data, allowing participants’ perspectives rather than our own clinical assumptions to guide the analytic process. All authors engaged in regular reflexive discussions to critically examine how our perspectives may have influenced the research process and the interpretation of findings [21]. NVivo 15 software was used to support data management and analysis.

The research team comprised professionals from various disciplines and clinical backgrounds, all female with experience in qualitative studies. LS (PhD) is an occupational therapist working in geriatric hospital care. NF (PhD) is a physiotherapist with experience in primary healthcare. CS (PhD) is a nurse specialised in the care of older adults, with experience in geriatric hospital care and care homes. A-MB (PhD) is a nurse specialised in primary healthcare nursing with experience of home healthcare. A-KW (PhD) is a physiotherapist with experience in geriatric hospital care and extensive experience in aging research.

## Results

Of the 24 participants, 12 took part in the *Simple exercise programme* and 12 in the *Comprehensive exercise programme*. Participants were aged 78–92 years (median: 85.5). The majority were women (67%) and lived alone (70.8%). Causes of hospital admission and baseline frailty levels varied across the sample. Length of hospital stay ranged from 4 to 8 days (median: 7 days), and the number of exercise sessions completed during hospitalisation also varied, with a median of 5 sessions. Further details on participant characteristics and the two intervention groups are presented in Table 2.

**Table 2:** Characteristics of the participants

Characteristic	Simple exercise programme *n* = 12	Comprehensive exercise programme *n* = 12	Total sample *n* = 24
*Demographic and clinical data*			
Age, median (min–max), years	86.5 (78–92)	83.5 (78–90)	85.5 (78–92)
Women, *n* (%)	10 (83.3)	6 (50)	16 (66.7)
Living alone, *n* (%)	9 (75)	8 (66.7)	17 (70.8)
Main cause of admission, *n* (%)			
Respiratory and circulatory disorders	4 (33.3)	4 (33.3)	8 (33.3)
Musculoskeletal disorders and injuries	3 (25)	4 (33.3)	7 (29.2)
Genitourinary and infectious disorders	2 (16.7)	3 (25)	5 (20.8)
General symptoms/deconditioning	3 (25)	1 (8.3)	4 (16.7)
CFS, median (min–max)	4.5 (4–7)	5 (3–5)	5 (3–7)
SPPB at admission, Median (min–max)	3 (1–12)	5.5 (1–12)	3 (1–12)
Length of hospitalisation, Median (min–max), days	6 (4–8)	7 (5–8)	7 (4–8)
Number of completed training sessions, median (min–max)	7.5 (2–12)	5 (3–7)	5 (2–12)

*CFS* Clinical Frailty Scale, *SPPB* Short Physical Performance Battery

**Fig.1:**
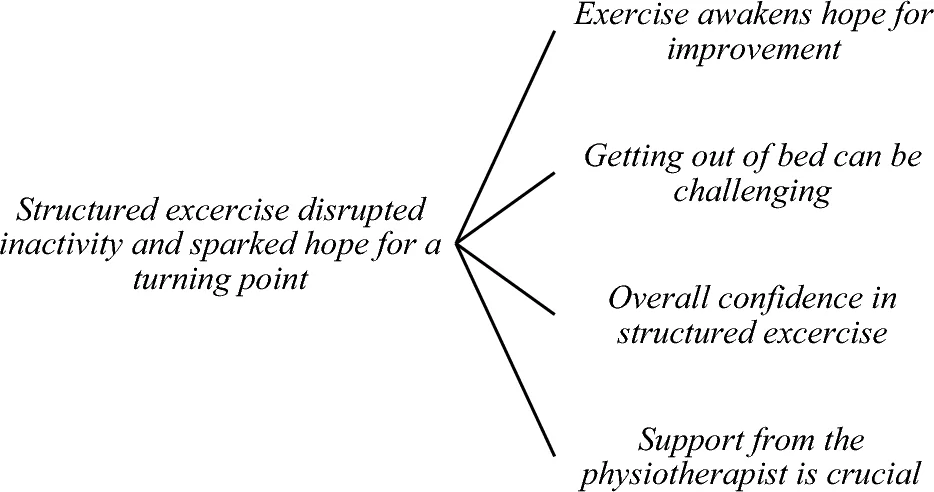
Themes representing older patients’ experiences of the two in-hospital exercise programmes

The participants’ experiences of engaging in one of the two exercise programmes during hospitalisation are described through the overarching theme: “Structured exercise disrupted inactivity and sparked hope for a turning point, and its four subthemes, as shown in Fig. 1

### Structured exercise disrupted inactivity and sparked hope for a turning point

Participants described the exercise sessions as a meaningful interruption to the prolonged inactivity that characterised most of their hospital stay, during which time was largely spent in bed. The exercise also provided a change of environment and social interaction that they looked forward to. The physiotherapist’s support was described as essential, and encouragement—sometimes experienced as a *“push”—*was important for motivating participants to get out of bed.

Overall, participants expressed confidence in both exercise programmes, noting that the sessions provided a balanced amount of exercise. However, some challenges were described, such as the structure and frequency of the sessions, as well as missed sessions due to not feeling well or undergoing medical examinations.

Engagement in exercise appeared to ignite an inner spark among participants, who described gaining hope for improvement. Starting the exercise and having the opportunity to progress was seen as a turning point—a chance to slow down physical decline, maintain or enhance physical function, and regain the ability to perform valued everyday activities.

### Exercise awakens hope for improvement

Participating in the exercise sessions was described by participants as igniting an inner spark and fostering hope that recovery was possible. The period prior to hospitalisation was often portrayed as a time of increasing passivity and declining health. They had stopped engaging in previously routine activities, such as walking, and had been unable to initiate any exercise on their own. Being given the opportunity to participate in exercise during their hospital stay was described by one participant: *“But now I’ve got a bit of a spark here, because I can feel how good it is to move and push myself a little… my body was stiff as a board… and now it’s loosened up a bit.”* (Woman, 86, Interview 12).

Participants described the exercise as a potential turning point and a starting place for resuming physical activity. One participant stated: *“But I must start somewhere, because I’ve just let everything slip away… I’ve reached the point now where I’ve gradually gotten worse and worse. And now it’s time to turn the whole ship around.”* (Man, 80, Interview 14).

The hospital setting was described as enabling participation in exercise, primarily because of accessibility. Participants emphasised that they did not need to go elsewhere to train and highlighted the significance of being given the chance to exercise, which also signalled that someone believed in them. One participant described the hospital-based exercise as a final “lifeline” and possibly a last chance for improvement.

Exercise was viewed positively in contrast to the general focus on illness and deterioration during the hospital stay, becoming a welcomed counterbalance. As one participant put it: *“Yes, it feels motivating to do something… there’s something positive about it [the exercise], not just all the negativity and feeling unwell. So, I think this counterbalance is needed.”* (Woman, 85, Interview 22).

Participants experienced positive feelings during and after the exercise sessions. Their positive perception of the exercise was strengthened when noticing physical improvements. Several participants expressed reduced stiffness, less pain, and improved circulation in their feet. They also described reduced muscle fatigue and increased physical strength. For example: *“In the beginning it was a bit shaky… with lactic acid in the thigh muscles after three or four rounds [referring to sit-to-stand exercises]. But it got better and better.”* (Woman, 92, Interview 18) . And another said: *“I’ve gotten stronger. I’ve mostly trained my knees, that was good.”* (Woman, 81, Interview 17). These tangible improvements reinforced the experience of a clear turning point. One participant described being able to perform more repetitions of an exercise during her hospital stay: “*I could suddenly just stand up. It was like ‘take your bed and walk.’ I mean, it was… it was an incredibly… a positive experience.”* (Woman, 80, Interview 9).

Participants emphasised that even small improvements could make a significant difference in their daily lives. One participant noted that simply being able to stand up on her own, a recurring exercise, would allow her to manage toilet visits independently. Maintaining or improving functions, such as strength and balance, and resuming activities, were key motivators for exercising. Participants focused on how they would manage once they returned home, expressing hope that they would be able to continue with their exercise programmes independently after discharge.

### Getting out of bed can be challenging

A recurring statement among participants was that without encouragement, they likely would have remained in bed. Participants described a natural resistance to getting out of bed when they did not feel well—particularly when energy was low and pain was present, which made mobilisation even more difficult. Outside of the exercise sessions, participants noted that there was often little to get up for and not much happened on the wards. Several expressed that time in the hospital felt slow and that they were grateful for the interruptions that the exercise sessions provided. The session helped time pass more quickly: *“It’s a bit of a break from the monotony.”* (Man, 89, Interview 10). The sessions introduced a sense of enjoyment and engagement during the day. Another participant said: *“This has been good and fun actually*—*instead of just lying on the bed and spinning around, so it’s been good…”* (Man, 85, Interview 3).

Participants also described how the exercise helped them leave their patient rooms, which contributed to a welcome change of environment. While the corridor could sometimes be perceived as noisy and chaotic, getting out provided opportunities for social interaction. Social interaction with the physiotherapist was appreciated by several participants. One participant expressed the value of both environmental change and social contact: *“Lying like this makes you very passive. It’s quite nice to meet someone and go down and do something in another room and things like that too. It’s positive. Social, or whatever you want to call it, not just lying flat in bed, I think.”* (Woman, 81, Interview 5).

The extent to which participants exercised independently varied. Some continued with exercises in bed that they were used to doing every morning at home. Others took initiative to perform exercises from their in-hospital exercise programme, even on weekends when physiotherapists were not present. However, some found the exercise programmes quite demanding and felt they lacked the energy to extend the training sessions on their own.

### Overall confidence in structured exercise

Throughout the interviews, participants expressed confidence that the exercise programme and movement were beneficial for them. However, participants’ experiences varied in relation to the specific programme they followed. In the *Simple programme*, which comprised four daily sessions, some individuals expressed that the frequency felt excessive and that a reduced number of sessions would have been sufficient. While the repetition of the same exercises was occasionally described as monotonous, others appreciated the familiarity it provided. In contrast, participants in the *Comprehensive programme,* involving two sessions per day, tended to describe the frequency as more manageable, contributing to a more positive perception of the programme’s structure.

Regarding the exercises themselves, participants were generally satisfied. Those in the *Simple programme*, which consisted of sit-to-stand exercises and walking, sometimes wished for more variety. While the exercises could feel repetitive, it was also noted that simple exercises were beneficial if they were to continue exercise at home. Some questioned whether standing up and walking truly qualified as exercise, while others emphasised the functionality and usefulness of these everyday movements, noting that no special equipment was required. One participant said: *“And you don’t need a lot of extra stuff. You always have access to a bed or a chair.”* (Woman, 85, Interview 22).

Participants in the *Comprehensive programme*, which included a wider range of exercises, expressed interest in adding even more activities, such as arm exercises. Some reflected on the limited availability of similar equipment at home, although a few had equipment or planned to acquire it. The *Comprehensive programme* was described as somewhat challenging, particularly due to the balance exercises. While these exercises could feel uncomfortable at times, they were generally appreciated as participants emphasised the importance of improving balance to reduce the risk of falls. One participant said: *“Yes, well, that’s really the whole point, I’d say – to prevent falls. It’s about being able to stand up and sit down.”* (Woman, 85, Interview 23). Among those in the *Simple programme*, perceptions of exertion varied—from easy to demanding: *“At first, I thought the demands seemed too high. Today, whether it was the second or third time, I felt how much… how much progress I had made.”* (Woman, 82, Interview 7).

The length and content of the sessions were generally described as providing a balanced amount of exercise, leaving participants motivated to exercise again. One participant described it: *“Otherwise, when you’ve trained, you’ve maybe been out a bit too long and pushed too hard. Here, it’s just the right amount.”* (Woman, 85, Interview 22).

Participants expressed both a desire for increased and reduced exercise. In both groups, some reported muscle soreness as a positive sign, indicating that the exercise was effective. However, there were reflections on the short duration of the hospital stay and the limited number of sessions completed, questioning whether the exercise could make a difference.

There were also occasions when planned sessions were missed. Sometimes participants did not feel well enough to exercise, due to fatigue, dizziness, or pain. One participant said: *“Yes, I’ve declined once or twice. But that was because I was tired for other reasons. But most of the time I’ve done it.”* (Woman, 78, Interview 2). Another reason for missed sessions was being away for medical examinations. Participants found it problematic to be away for exercise, as it meant they were unavailable to other medical staff who wanted to reach them—even though staff knew they were exercising and would return. One participant said: *“If they see I’m not there, they’ll come back… I thought… Maybe it was a bit disruptive, that I wasn’t available when they came and wanted me.”* (Man, 88, Interview 24).

### Support from the physiotherapist is crucial

The support participants received from the physiotherapist leading their exercise sessions was described as significant, and at times even essential for the exercise to take place. It was often difficult for participants to initiate exercise on their own. One participant expressed: *“If no one came into my room and said, ‘Now you’re going to do five sit-to-stands,’ would I get out of bed when I’m tired and do it? No! But if someone comes and says, ‘Do it.’ Yes, then I do it.”* (Woman, 85, Interview 8). Encouragement and what participants described as *“a push”*, was seen as helpful in breaking the pattern of staying in bed: *“Of course it’s positive to get that support and push and everything. Instead of just lying here waiting to get better. So… No, but it’s positive, I must say. Absolutely. It is. Yes.”* (Woman, 78, Interview 21).

Participants expressed a strong trust in the physiotherapists’ competence, believing that they knew what they were doing and that the exercise was tailored to individual needs. Feeling capable of performing the exercises was described as highly meaningful. One participant said:

*“And then it’s nice to feel that you can manage to do things. That’s psychologically important, I think.”* (Woman, 85, Interview 22). Having the physiotherapist nearby during exercise also created a sense of safety, especially when their balance was challenged.

Some participants noted that receiving praise during the sessions felt encouraging and gave a sense of satisfaction even when they did not think what they had done was particularly remarkable. One participant said: *“Yes, but it was fun that I could manage it, I thought. And I got a bit of praise and so on [laughs]. You’re never too old for that. Right?”* (Woman, 86, Interview 12).

The nature of the relationship with the physiotherapist was also highlighted as an important aspect of the exercise experience. Participants valued having an equal relationship rather than a hierarchical one. One participant said: *“They haven’t been domineering. It’s been on equal terms, and that feels really good.”* (Woman, 78, Interview 2). Physiotherapists were often perceived as positive, and it was important for participants to feel involved in the decisions about their exercise. One participant expressed: *“Yes, but just that he’s very positive, and checks in on which parts I like best and such, so I feel like I get to be part of deciding what kind of exercise it will be.”* (Woman, 85, Interview 22). Participants also appreciated when the physiotherapist joked during the sessions, which brought positive energy: *“She was always cheerful, and we joked a bit here and there, yes, yes, so that was good! Fun!”* (Man, 91, Interview 1).

Several participants described the exercise as something they did together with the physiotherapist—as a shared activity. One participant said: *“We stood and bent forward and did squats, yes, that’s what we did. That’s what she and I did together.”* (Man, 91, Interview 1).

Because they spent repeated time with the physiotherapist, there was also room to ask questions that arose during their hospital stay. These questions did not always concern the exercise itself, but could relate to practical concerns, such as how to get up from the floor after a fall or how to manage stairs at home.

Both the support that made them feel safe and the *“push”* to get started with exercise were described as crucial aspects for completing the sessions. Several participants reflected that it might be difficult to continue exercising independently after discharge without the support of the physiotherapist. One of the participants said: *“But I can’t really picture how I would be able to exercise at home. I mean, I don’t really know what it is… or what I’m supposed to do.”* (Woman, 80, Interview 9).

## Discussion

This study explored older patients’ experiences of two in-hospital exercise programmes. The structured exercise were perceived as helpful in breaking the pattern of inactivity and sparked hope for a turning point. Participants described getting out of bed as challenging, highlighting the value of structured exercise and a hospital environment that promotes movement. Having a planned activity was described as a facilitator for getting out of bed and the support from the physiotherapist was perceived as crucial. Promoting physical activity during hospital stays is essential, as inactivity may lead to loss of muscle mass, reduced mobility, and increased risk of rehospitalisation [1–3].

The exercise itself was described as a counterbalance to illness and physical decline. In clinical settings, medical examinations are often prioritised, and mobilisation is frequently neglected [22]. Although medical care must remain a priority, the importance of physical activity and exercise should be emphasised. Participants described that the training sessions could ignite a spark and foster a sense of hope and belief in recovery during hospitalisation. Similar findings were reported among care home residents, where participants experienced increased self-efficacy and renewed hope after engaging in a simple exercise programme [23]. These findings suggest that exercise can play a valuable role in strategies aimed at enhancing well-being among hospitalised older adults.

Although the participants expressed overall confidence in the exercise programmes, they also emphasised the need for greater individualisation, which aligns with findings from the previous study [7]. The repetitive nature of the *Simple exercise programme* was perceived as a limitation by some participants. However, at the same time, this repetition was seen as beneficial, as it may facilitate continued exercise after discharge. Participants appreciated that the programme required only a chair, a readily available tool, making it accessible for all patients. The *Simple programme* may, therefore, serve as a starting point for patients with a higher degree of frailty, particularly those with low muscle strength and limited endurance.

The importance of balance training during hospitalisation was highlighted, expressing a desire to feel stable and a fear of falling. Fear of falling is a concern well-documented in previous research [24, 25 ]. Furthermore, pre-existing perceptions of exercise appear to influence patients’ motivation to exercise [26]. If these exercise interventions were to be implemented among patients with low motivation, complementary strategies, such as goal setting, fall prevention measures, and education, on the benefits of movement during hospitalisation may enhance the effectiveness of the exercise programmes [26].

Consistent with previous research, the physiotherapist played a crucial role in ensuring that the exercise sessions were carried out [9, 26, 27 ]. Participants expressed strong trust in the physiotherapist’s competence and valued the social interaction that the sessions provided. This underscores the importance of staff expertise in delivering exercise programmes and suggests that, if other professional groups are to lead sessions, additional training may be needed. Future studies should address this issue contingent upon evidence and appropriateness of these programmes for implementation.

Concerns emerged regarding the ability to continue the exercise programme independently after discharge. Initiating and maintaining exercise routines without professional support can be particularly challenging for frail older adults [28, 29 ]. These concerns are consistent with previous research on rehabilitation services, which indicates that older adults returning home after hospitalisation often require support tailored to their current functional status. Exercise programmes initiated during hospitalisation may need to be adapted to fit the home environment [24]. In light of these findings, further research is warranted to explore how older adults who have been hospitalised perceive the continuation of home-based exercise following discharge, as well as what type of support may facilitate sustained engagement.

### Study limitations and strengths

Some methodological considerations should be acknowledged. The sample may have been affected by selection bias and participant homogeneity, as all participants were recruited from the PREV_FUNC trial at a single hospital. Being a patient in a hospital may entail a vulnerable situation and could influence how participants express themselves. To mitigate this, all interviews were conducted in a quiet and private room. Conducting the interviews in the hospital allowed participants to reflect shortly after completing the exercise sessions. Additionally, the hospital environment itself may have shaped participants’ responses; however, the interviewer (LS), being familiar with this setting through professional experience, sought to facilitate open dialogue.

In addition to considerations related to the interview context, aspects of sampling and transferability should also be considered. As participants had already agreed to participate in an exercise intervention, they likely represent a group with higher motivation and more positive attitudes toward exercise than the general population of hospitalised older adults. Purposive sampling based on age, sex, and group allocation (*Simple* or *Comprehensive exercise programme*) enabled the inclusion of varied perspectives and experiences [18]. Although frailty, physical performance, and medical conditions were not used as inclusion criteria, participants varied in these characteristics, capturing a broad range of experiences. The detailed description of the study context, participant characteristics, and data collection and analysis procedures provides a solid basis for readers to assess the transferability of the findings to similar settings [14].

The authors acknowledge that the analysis was informed by their pre-understandings, shaped by professional backgrounds in health and ageing. To ensure that the findings reflected the participants’ perspectives, the data were analysed thoroughly and iteratively using an open coding approach. Reflexivity was embedded throughout the analytic process, with LS and NF engaging in an ongoing dialogue about emerging interpretations, which were regularly discussed within the full research team. The involvement of multiple researchers with varied expertise contributed to the richness and depth of the findings, while continuous reflection on personal preconceptions supported analytic rigor and trustworthiness [30, 31 ]. Trustworthiness was further supported through transparent reporting of each step of the analysis [14].

## Conclusions

In conclusion, from the patients’ perspective, in-hospital exercise programmes appear feasible and promote physical activity by disrupting inactivity and fostering hope for improvement. Responsiveness to individual needs is essential to support benefits, and the programmes can provide a foundation for supporting physical activity in geriatric care. Integrating these insights with forthcoming PREV_FUNC trial outcomes can guide future clinical practice.

## Supplementary Information

Below is the link to the electronic supplementary material.

Supplementary file1 (PDF 555 KB)

Supplementary file2 (DOCX 22 KB)

## Data Availability

The data supporting the findings of this study are not publicly available due to the permission given from the Swedish Ethical Review Authority.
