# Supplementary material for: Older patients’ experiences of two exercise programmes in a geriatric ward: a qualitative study
Source: Eur Geriatr Med. 2026 Aug 5;17(5):2945–54. doi: 10.1007/s41999-026-01569-w (PMC13755044; doi:10.1007/s41999-026-01569-w)
Supplement: Supplementary file 2 [file 41999_2026_1569_MOESM2_ESM.docx]

# **Supplementary material**

# **Interview guide – Individuals who participated in the training program**

-Can you tell me about your exercise habits before your hospital stay?

-How did you experience exercising at the hospital/at home?

-What did you find good and less good about exercising at the hospital?

-Was there anything you found enjoyable or pleasurable about the training?

-Did you feel that the training was meaningful? In what way was it meaningful to you?
If no: What could have made it feel more meaningful to you?

-How was your motivation to exercise? What increased your motivation to train? / What decreased your motivation?

-What aspects of the training felt relevant to you? Was there anything that didn’t feel relevant?

-How physically demanding was the training? How did you experience it?

-How did you perceive the structure of the training in terms of number of sessions and session length? Would you have preferred it to be different in any way?

-What made you feel safe during the training? Was there anything that made you feel unsafe?

-Do you have any suggestions for how the training could be improved? / Was there anything you wish had been different?

-In what way did you feel supported during the training? Would you benefit from additional support? What kind of support would have helped you?

-How was it to exercise in the hospital environment/at home? What opportunities or obstacles did the environment present when you were doing the training?

-What do you take with you from the training you’ve done here?

-How do you think the training here will affect your future exercise habits?

-Have you done any exercise on your own outside of the training program while you’ve been on the ward/at home? What did you do? How was that experience?

-How do you feel the training has affected your health? How do you feel it has influenced your physical ability / activity level / mood / sleep?

-Is there anything else you would like to add before we finish?

-Do you have any questions for me?

**Examples of Follow-Up Questions**
-Can you give an example?
-Can you tell me more?
-What does that mean to you?

# **Intervjuguide – personer som har erhållit träning i projektet**

-Berätta om dina träningsvanor innan sjukhusvistelsen?

-Hur har du upplevt att det har varit att träna på sjukhuset/hemma?

-Vad tycker du har varit bra och mindre bra med att träna på sjukhuset/hemma?

-Var det något som du tyckte var roligt / lustfyllt med träningen?

-Upplevde du att träningen var meningsfull? På vilket sätt var den meningsfull för dig?
Om nej: Vad hade kunnat göra att den upplevdes mer meningsfull för dig?

-Hur har din motivation varit till att träna? Vad ökade din motivation att träna? / Vad minskade din motivation att träna?

-Vad i träningen upplevdes relevant för dig? Var det något som inte kändes relevant?

-Hur ansträngande var det att träna? Hur upplevde du det?

-Hur upplever du träningens upplägg avseende antal pass och passens längd? Hade du önskat att den var på något annat sätt?

-Vad gjorde att du kände dig trygg under träningen? Var det något under träningen som gjorde att du inte kände dig trygg?

-Har du några förslag på hur träningen kan förbättras? / Hade du önskat att något var annorlunda?

-På vilket sätt upplever du att du har fått stöd under träningen? Hade du varit hjälpt av något mer stöd? Hur skulle det stödet vara utformat för att hjälpa dig?

-Hur har det varit att träna i sjukhusmiljön/hemma? Vad har det funnits för möjligheter/hinder i miljön när du skulle utföra träningen?

-Vad tar du med dig från träningen som du har gjort här?

-Hur tror du att träningen här kommer påverka din framtida träning?

-Har du tränat något själv nu utöver träningsprogrammet när du har varit på avdelningen/hemma? Vad har du gjort då? Hur har det varit?

-Hur upplever du att du att träningen har påverkat din hälsa? Hur upplever du att träningen har påverkat din fysiska förmåga / aktivitetsförmåga / sinnesstämning / sömn?

-Är det något annat som du önskar tillägga före vi avslutar? -Har du några frågor till mig?

Stort tack för att du ville dela dina erfarenheter och tog dig tiden att vara med!

Exempel på följdfrågor Kan du ge något exempel? / Kan du beskriva det mer? / Kan du berätta mer? / Hur menar du då? / Vad betyder det för dig?
